# Facile and Rapid Electrochemical Conversion of Ni into Ni(OH)_2_ Thin Film as the Catalyst for Direct Growth of Carbon Nanotubes on Ni Foam for Supercapacitors

**DOI:** 10.3390/nano12213867

**Published:** 2022-11-02

**Authors:** Sheng-Hung Kao, Krishnan Shanmugam Anuratha, Sung-Yen Wei, Jeng-Yu Lin, Chien-Kuo Hsieh

**Affiliations:** 1 Department of Materials Engineering, Center for Plasma and Thin Film Technologies, Ming Chi University of Technology, New Taipei City 24301, Taiwan; 2 Department of Chemical and Materials Engineering, Tunghai University, Taichung City 407224, Taiwan; 3 R&D Lab, SulfurScience Technology Co., Ltd., New Taipei City 24301, Taiwan

**Keywords:** cyclic voltammetry, Ni(OH)_2_, CNTs, EDLC, supercapacitors

## Abstract

In this paper, a facile and rapid aqueous-based electrochemical technique was used for the phase conversion of Ni into Ni(OH)_2_ thin film. The Ni(OH)_2_ thin film was directly converted and coated onto the network surface of Ni foam (NF) via the self-hydroxylation process under alkaline conditions using a simple cyclic voltammetry (CV) strategy. The as-formed and coated Ni(OH)_2_ thin film on the NF was used as the catalyst layer for the direct growth of carbon nanotubes (CNTs). The self-converted Ni(OH)_2_ thin film is a good catalytic layer for the growth of CNTs due to the fact that the OH^−^ of the Ni(OH)_2_ can be reduced to H_2_O to promote the growth of CNTs during the CVD process, and therefore enabling the dense and uniform CNTs growth on the NF substrate. This binder-free CNTs/NF electrode displayed outstanding behavior as an electric double-layer capacitor (EDLC) due to the large surface area of the CNTs, showing excellent specific capacitance values of 737.4 mF cm^−2^ in the three-electrode configuration and 319.1 mF cm^−2^ in the two-electrode configuration, at the current density of 1 mA cm^−2^ in a 6 M KOH electrolyte. The CNTs/NF electrode also displayed good cycling stability, with a capacitance retention of 96.41% after 10,000 cycles, and this the excellent cycling performance can be attributed to the stable structure of the direct growth of CNTs with a strong attachment to the NF current collector, ensuring a good mechanical and electrical connection between the NF collector and the CNTs.

## 1. Introduction

Electric double-layer capacitors (EDLCs) are one type of important supercapacitors (SCs) known for their rapid charge–discharge performance, long cycle ability, high safety, and high specific power density [1]. Carbon-based nanomaterials have attracted intense interests as electrode materials for EDLCs due to their remarkable high surface area, chemical stability, and electrical conductivity [2]. Among carbon-based nanomaterials, carbon nanotubes (CNTs) are a class of one-dimensional (1D) tubular carbon nanomaterials with a graphitic structure; they are favored for their unique ballistic electronic transport behavior over long tube ranges, without electronic scattering [3]. This property allows 1D CNTs to carry high densities of currents without energy dissipation, promoting them as candidates for EDLCs. In addition, the most effective strategy for using CNTs to achieve high-performance EDLCs is directly growing CNTs on the current collector. There are many advantages of this method [4,5,6]; for instance, the absence of binders can effectively reduce the interfacial resistance between CNTs and the current collector. CNTs uniformly grown onto the surface of a current collector avoids the agglomeration issue and therefore, maximizes the utilization of the surface area of the CNTs. Additionally, owing to their ballistic property, CNTs act as high-speed channels to minimize energy loss. However, the directly grown CNTs still remain challenging, especially regarding the metallic current collector commonly used as a substrate.

Considering the synthesis of CNTs, the most common method for their direct growth is thermal chemical vapor deposition (CVD) [7,8]. In such a process, the catalytic metallic particles used for supporting CNT growth are often obtained from metallic thin films nucleated during the high-temperature annealing stage. However, the high-temperature annealing stage leads to the cross diffusion between the catalytic metallic thin films and the metallic substrates, creating a bottleneck for the direct growth of the CNTs onto the metallic substrates. Currently, there are few studies reporting the successful growth of CNTs onto metallic substrates via an oxide buffer layer between the catalytic metallic thin films and metallic substrates. For instance, Lee et al. grew CNTs directly on stainless steel mesh via the CVD method, with a diffusion barrier of Al_2_O_3_ layer between the Fe catalytic layer and stainless-steel substrate [9]. However, the oxide buffer layer, exhibiting intrinsic high resistance, deteriorated the electrochemical performance.

Ni(OH)_2_ was found to be the catalytic nanomaterial for the synthesis of CNTs at a low temperature of 450 °C via the CVD method, as was reported by Zeng’s group [10]. Ni(OH)_2_ can be easily fabricated via the hydrothermal method, the solvothermal method, and electrochemical treatment [11,12,13,14]. In regards to large-scale production for industrial fabrication, electrochemical treatments are currently widely used in this field. Additionally, the transition metal can be easily converted to metal hydroxide via redox reactions under the alkaline aqueous solution with electrochemical treatment, resulting in ion-exchange reactions [13]. Therefore, we herein report the synthesis of an Ni(OH)_2_ thin film, which was self-converted from an Ni foam (NF) substrate via the facile cyclic voltammetry (CV) method, to obtain the Ni(OH)_2_/NF and the subsequent direct growth of CNTs on NF by using the as-converted catalytic Ni(OH)_2_ thin film via a low-temperature CVD for the fabrication of a CNTs/NF electrode. By using this self-converted process via the electrochemical CV strategy, Ni(OH)_2_ thin films can be uniformly formed and coated onto the surface of NF networks for the subsequent effective distribution of growing CNTs to obtain the CNTs/NF electrode and achieve high-performance EDLC.

## 2. Experimental Method

### 2.1. Fabrication of Ni(OH)_2_ Thin Films and CNTs/NF Electrodes

All NF substrates (110 ppi, 1.7 mm, HOMYTECH Co., Ltd., Taoyuan, Taiwan) were cut into 1 × 1 cm^2^ pieces and each piece was ultrasonically cleaned, in sequence, using acetone, deionized (DI) water, and ethanol for 15 min each. The CV-converted Ni(OH)_2_ thin films were synthesized using a potentiostat/galvanostat device (Autolab PGSTAT 302N (Metrohm Autolab B.V., Utrecht, The Netherlands)) in a three-electrode configuration at a controlled temperature of 25 °C under ambient pressure. A Pt foil and a saturated calomel electrode (SCE) were used as the counter and reference electrode, respectively. The NF substrate was initially subjected to a CV treatment in a plating bath containing 3 M KOH and scanned from 0 to 0.6 V_SCE_, with a scan rate of 200 mVs^−1^ for 50 cycles, to facilely and rapidly obtain the Ni(OH)_2_ thin films coated onto the surface of NF networks.

The as-obtained Ni(OH)_2_/NF sample was washed with DI water and dried at room temperature for 30 min. Subsequently, the Ni(OH)_2_/NF sample was loaded into the quartz tube furnace thermal CVD chamber for the growth of CNTs. In brief, the furnace was heated to 450 °C under an Ar atmosphere. Prior to the CVD process, the quartz tube was pumped to 10^−2^ torr in order to confirm the complete removal of air. Then, a C_2_H_2_ gas of 10 sccm was introduced into the reaction tube for 20 min for the direct growth of CNTs onto the surface of NF networks, and then subsequently cooled to room temperature under an Ar atmosphere to remove the CNTs/NF electrode. The furnace pressure was maintained at 20 torr throughout the entire CVD process.

### 2.2. Characterization and Measurements

The prepared samples were characterized using scanning electron microscopy (SEM, JEOL, JSM-6330F (Japan Electron Optics Laboratory Co., Ltd., Akishima City, Japan)), high-resolution transmission electron microscopy (HR-TEM, JEOL, JEM-2100F (Japan Electron Optics Laboratory Co., Ltd., Akishima City, Japan)), Raman spectra (LABRAM HR 800 UV, equipped with a 514 nm laser source (Horiba, Ltd., Kyoto, Japan)), and X-ray photoelectron spectroscopy (XPS, PHI Quantera 5000 VersaProbe III (ULVAC-PHI Inc., Kanagawa, Japan)).

Electrochemical measurements were performed using three techniques—CV, galvanostatic charge/discharge (GCD), and electrochemical impedance spectroscopy (EIS)—using the same potentiostat/galvanostat electrochemical workstation (Autolab PGSTAT302N (Metrohm Autolab B.V., Utrecht, The Netherlands)) for the 6 M KOH aqueous electrolyte.

## 3. Results and Discussion

### 3.1. Characteristics

**Scheme 1:**
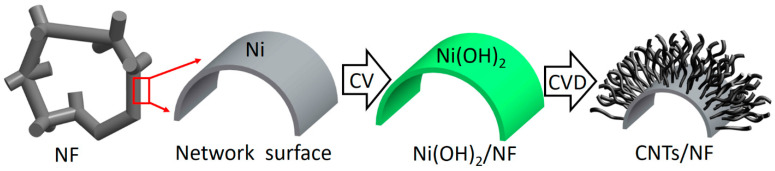
Schematic of the fabrication procedure of a CNTs/NF electrode.

**Figure 1:**
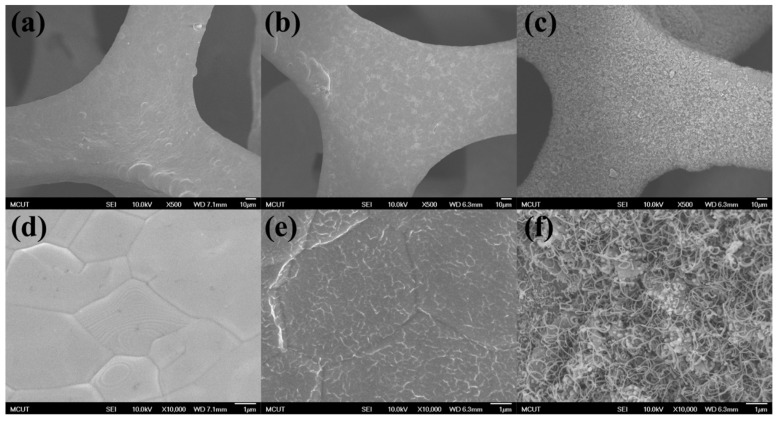
Low-magnification FESEM images of (**a**) NF, (**b**) Ni(OH)_2_/NF, and (**c**) CNTs/NF, respectively. High-magnification FESEM images of (**d**) NF, (**e**) Ni(OH)_2_/NF, and (**f**) CNTs/NF, respectively.

The schematic representation of the fabrication procedure of the CNTs/NF electrode is illustrated in Scheme 1. Figure 1a–c and Figure 1d–f show the low- and high-magnification FESEM images of NF, Ni(OH)_2_/NF and CNTs/NF, respectively. As seen from the high-magnification FESEM images, Figure 1d displays the clearly visible Ni grain boundaries (GBs) of the surface of the NF network. Figure 1e shows that the Ni(OH)_2_ thin film, uniformly formed and coated onto the surface of the Ni network, exhibits numerous small wrinkles, which means that the Ni(OH)_2_ thin film displays the characteristics of a nanowall structure. In addition, Ni GBs still can be seen, revealing that the Ni(OH)_2_ thin film is very thin. Figure 1f shows the dense and uniform CNTs well grown onto the surface of NF via the CVD process. It is worth noting that the method of directly growing the CNTs avoids the agglomeration issue and provides an open microstructure to facilitate the diffusion of electrolytes; thus, the electrolytes can be easily diffused through them, and thus, better electrochemical performance is expected. Without the use of a binder, the interfacial resistance between the CNTs and the NF collector demonstrates outstanding electrical properties.

**Figure 2:**
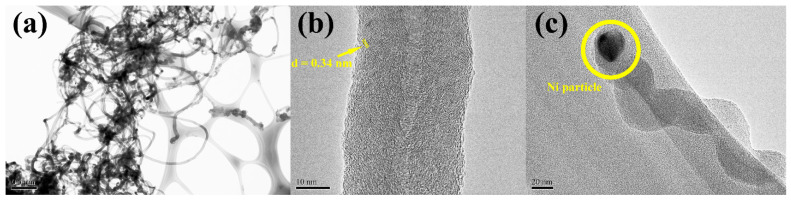
(**a**) Low-magnification TEM image of CNTs, and HRTEM images of (**b**) body and (**c**) head of CNT.

Figure 2a shows the TEM image of CNTs with a uniform external diameter of around 30 nm. Figure 2b,c shows the HRTEM images of the CNT body and head, respectively. As seen from Figure 2b, the tubular nanostructure and the cup-stacked graphitic layers are clearly seen in the cup-stacked CNT; the distance to the graphitic interlayer is around 0.34 nm. In general, the concentric CNTs are the result of the smooth wall surface of the basal plan and the poor surface accessibility for the ions. The cup-stacked carbon layers are the edge sites of cup-stacked CNTs, the edge sites with a large proportion of exposed open edges present on the outer surface, leading to enhanced accessibility for the ions and the promotion of electrochemical performance [15]. In fact, the capacitance of the edge plane of carbon materials is much higher (10–100 times) than that of the basal plane [16,17].

The Ni nanoparticle in the head of CNT is around 20 nm, as shown in Figure 2c. Chemically, the reduction of divalent nickel to metallic Ni is realized in the presence of C_2_H_2_, according to speculated Equations (1) and (2):

C_2_H_2_ → 2C + H_2_ (1)

Ni(OH)_2_ + H_2_ → Ni + 2H_2_O (2)

In addition, H_2_O acts plays a key role in controlling the growth of CNTs, according to previous reports [18,19,20]. During the CVD reactions, the amorphous carbon coating onto the catalysts will reduce their activity, creating a suitable oxidizer that would selectively remove amorphous carbon without damaging the graphitic CNTs at the low growing temperature. Thus, H_2_O acts in promoting and preserving catalytic activity. In our case, CNTs were grown using C_2_H_2_ as the carbon source, and the decomposed H_2_ reacted with Ni(OH)_2_ to produce a crucial amount of water vapor to influence the synthesis of CNTs. Therefore, the reactions can be combined, according to Equation (3):

Ni(OH)_2_ + C_2_H_2_ → Ni + 2H_2_O + 2C_CNTs_ (3)

**Figure 3:**
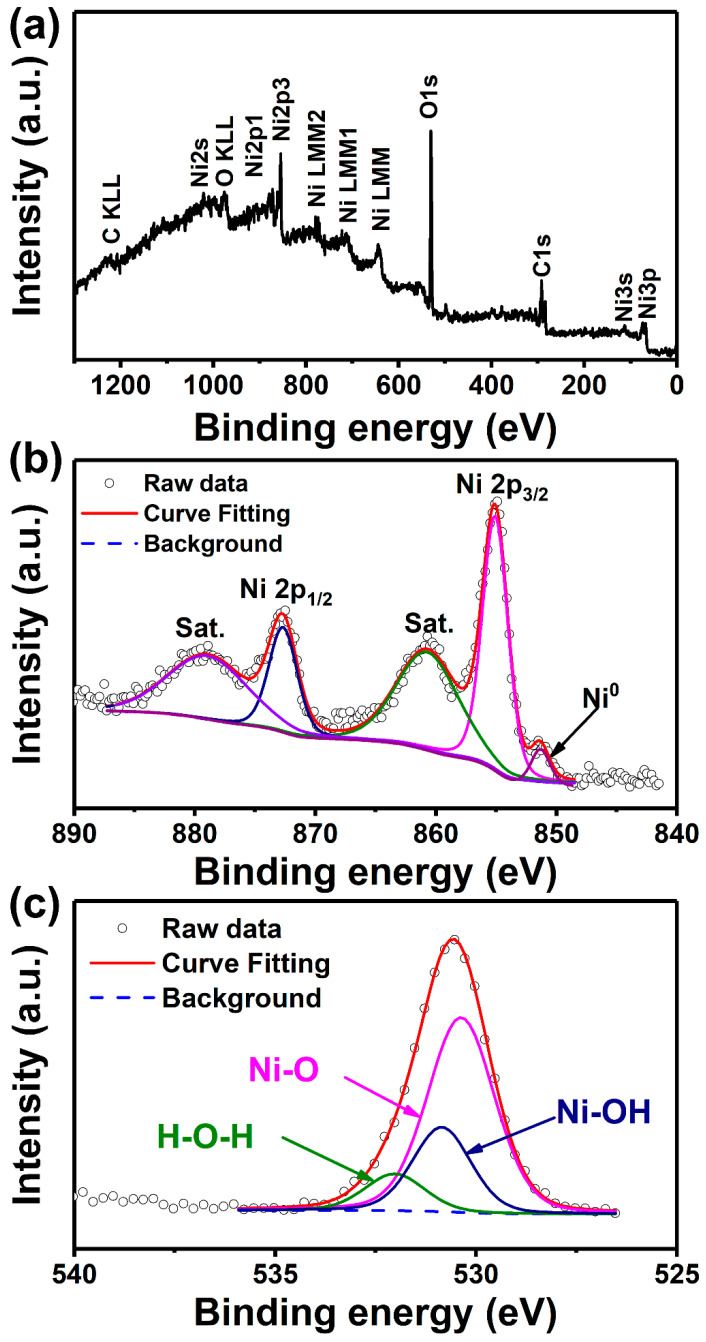
XPS of Ni(OH)_2_ thin film: (**a**) survey spectrum; (**b**) Ni 2p; (**c**) O1s curves.

XPS is employed to investigate the chemical composition and bonding states of the prepared Ni(OH)_2_/NF. Figure 3a shows the full-survey of the XPS spectrum, including the peaks of C 1s, O 1s, Ni 2p_1/2_, and Ni 2p_3/2_. Figure 3b presents the high-resolution spectrum of the Ni 2p region; the curve was analyzed by using a Gaussian–Lorentzian peak after completing the Shirley background correction. The Ni^0^ at 852.5 eV was within that previously report for the Ni metal peak value of 852.4 ± 0.4 eV. The two major peaks of Ni 2p_1/2_ at 872.9 eV and Ni 2p_3/2_ at 855.3 eV, along with their two satellite peaks located at 879.4 eV and 861.0 eV, comprise the Ni 2p peak, as shown in Figure 3b, and the peaks of Ni-O, Ni-OH, and H-O-H, located at 530.4 eV, 531.1 eV, and 532.1 eV, are shown in Figure 3c, which confirms the distinct characteristics of Ni(OH)_2_, as revealed in previous reports [13,14,21,22]. Additionally, the spin-energy separation of the two major peaks of Ni 2p_1/2_ and Ni 2p_3/2_ is 17.6 eV, which also further supports the presence of Ni(OH)_2_ [21]. According to the XPS analysis results, Ni ions would be self-converted, forming an Ni(OH)_2_ thin film coating onto the NF surface during the CV cycling and the application of voltage in the KOH aqueous solution, as shown below in Equations (4) and (5):

Ni → Ni^2+^ + 2e^−^ (4)

Ni^2+^ + 2OH^−^ → Ni(OH)_2_ (5)

**Figure 4:**
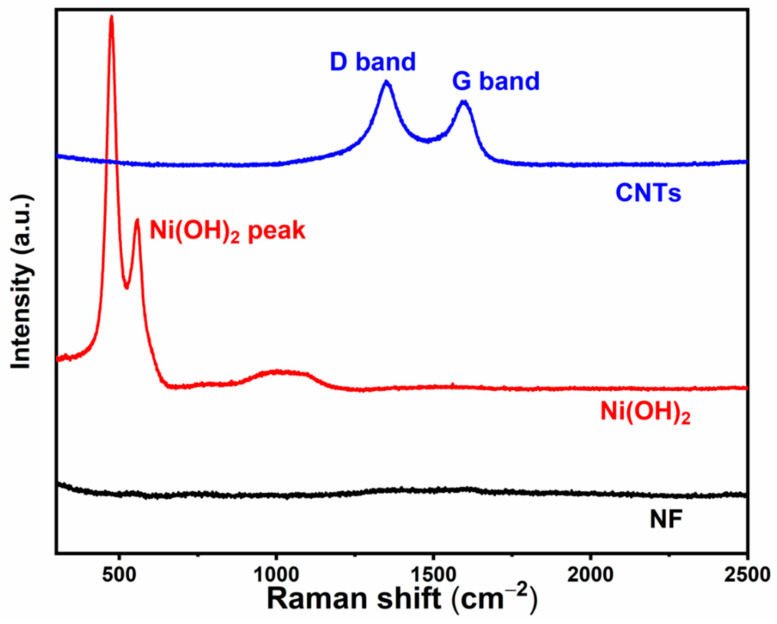
Raman spectrum of Ni(OH)_2_ thin film converted via the CV method (red line), and CNTs grown by the CVD process (blue line).

Figure 4 shows the Raman spectrum of the NF, the as-prepared Ni(OH)_2_/NF, and the CNTs/NF, respectively. The Raman pattern of the Ni(OH)_2_ thin film converted via the CV method (red line) shows two peaks located at 479 and 557 cm^−1^, which can be attributed to the symmetric Ni-OH stretching mode and the vibration of the Ni-O stretching mode of Ni(OH)_2_, respectively [21,23,24]. The Raman result of Ni(OH)_2_ is also consistent with the XPS spectrum. The Raman pattern of CNTs/NF shows two characteristic peaks located at 1360 and 1590 cm^−1^, which are the D band associated with the defects of carbon nanomaterials and the G band originated from the in-plane vibration of sp^2^-hybridized carbon atoms [25]. Further evidence of the uniform growth of CNTs can be observed from the disappearance of the two peaks of the Ni(OH)_2_ thin film, which have been completely consumed as Ni catalytic nanoparticles used for directly growing the CNTs. The in situ self-converted and formed Ni(OH)_2_ thin film was used as the catalyst layer for directly growing CNTs onto the NF surface, and the CNTs are in direct contact with the NF to fabricate the CNTs/NF electrode.

### 3.2. Electrochemical Properties

Both three-electrode and two-electrode configurations were used to measure the performance of the CNTs/NF as the supercapacitor electrode. The CV and GCD experiments were performed to investigate the areal specific capacities calculated according to Equations (6) and (7), respectively [26,27]:

(6) $$C_{s}=\frac{\int IdV}{2a\times \Delta V\times S}$$

where $C_{s}$,∫IdV, a, ΔV, and S are the areal specific capacitance, integrated area of the CV curve, area of the electrode, operating potential window, and the scan rate, respectively.

(7) Cs=Ia×(dVdt)

where $C_{s}$, I, a, and dVdt are the applied current density of GCD, area of the electrode, and the slope calculated from the discharge curve, respectively.

**Figure 5:**
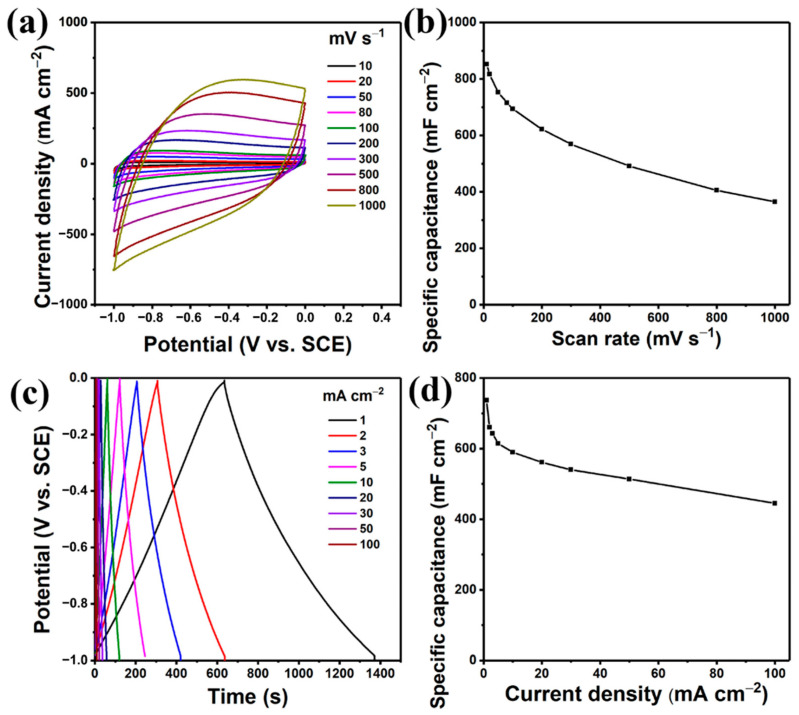
Electrochemical measurements of the CV and GCD curves using the three-electrode configuration: (**a**) CV curves of CNTs/NF electrode with different scan rates; (**b**) calculated areal specific capacitance values at different scan rates; (**c**) GCD curves of the CNTs/NF electrode with different current densities; (**d**) calculated areal specific capacitance values at different current densities.

Initially, the three-electrode configuration was carried out to examine the CV and GCD. The CNTs/NF was used as the working electrode, and a Pt foil and an SCE were used as the counter and reference electrode, respectively. Figure 5a shows the CV curves of the CNTs/NF electrode at different scan rates from 10 to 1000 mV s^−1^, in a potential window of 0 to −1.0 V_SCE_, and Figure 5b shows the areal capacitances calculated from the CV curves. Figure 5c shows the corresponding GCD curves examined under different current densities from 1 mA cm^−2^ to 100 mA cm^−2^ in a potential window of 0 to −1.0 V_SCE_, and Figure 5d shows the areal capacitances calculated from the GCD curves. The highest specific capacitance values derived from the CV and GCD curves of three-electrode configuration were 852.5 mF cm^−2^ at the scan rate of 10 mV s^−1^ and 737.4 mF cm^−2^ at the current density of 1 mA cm^−2^, respectively. Both the CV and GCD results also maintained good capacitance retentions of 42.8% and 60.4%, after increasing CV scan rate and GCD current density by 100 times.

**Figure 6:**
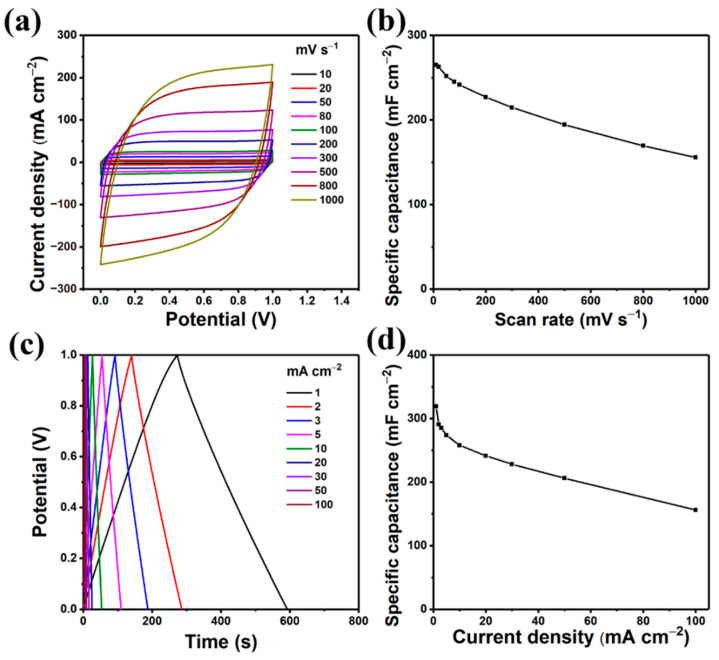
Electrochemical measurements of the CV and GCD curves of the two-electrode configuration: (**a**) CV curves with different scan rates; (**b**) calculated areal specific capacitance values at different scan rates; (**c**) GCD curves with different current densities; (**d**) calculated areal specific capacitance values at different current densities.

To further verify the capacitive properties of the CNTs/NF electrode, a two-electrode configuration was also used to examine the CV and GCD. Figure 6 shows the CV and GCD electrochemical performance of the CNTs/NF electrode, and Figure 6a shows the obtained CV curves at different scan rates from 10 to 1000 mV s^−1^ in a potential window of 0 to 1.0 V. The CV curves display the classic quasi-rectangular shapes and indicate the excellent EDLCs behavior of the carbon-based nanomaterials. Figure 6b shows the areal specific capacities calculated from the CV curves, and the areal capacitances obtained from the CV curves at different scan rates. It is worth noting that the two-electrode CNTs/NF cell not only exhibited high areal-specific capacitances, but also maintained a good capacitance retention. The two-electrode CNTs/NF cell showed a good areal specific capacitance of 265.1 mF cm^−2^ at 10 mV s^−1^ and maintained the capacitance retention of 58.6% at the super-fast scan rate of 1000 mV s^−1^ (265.1 mF cm^−2^ to 155.6 mF cm^−2^ at scan rates from 10 mV s^−1^ to 1000 mV s^−1^, with increasing the CV scan rate 100 times). Figure 6c displays the corresponding GCD curves measured under different current densities from 1 mA cm^−2^ to 100 mA cm^−2^; the symmetric and linear slopes of the GCD curves, without an IR drop, indicated its excellent EDLCs characteristics. Figure 6d shows the areal specific capacitance values calculated from the GCD curves at different current densities. The capacitance retention calculated from the GCD measurements is similar to that calculated from the CV curves. The two-electrode cell CNTs/NF exhibited the areal specific capacitance of 319.1 mF cm^−2^ at 1 mA cm^−2^ and still maintained a very good capacitance retention of 49.0% at a high current density of 100 mA cm^−2^ (319.1 mF cm^−2^ to 156.5 mF cm^−2^ at GCD current densities from 1 mA cm^−2^ to 100 mA cm^−2^, while increasing the GCD current density by 100 times). The details of GCD calculations are given in Table S1.

According to the CV and GCD results, the two-electrode CNTs/NF cell configuration showed good specific capacitance and capacitance retention. This phenomenon demonstrates that the open microstructure between the directly growing CNTs could promote the diffusion kinetics of the electrolyte, and the small interfacial resistance between the directly growing CNTs and the NF collector surface enhances the efficiency of the charge transfer.

**Figure 7:**
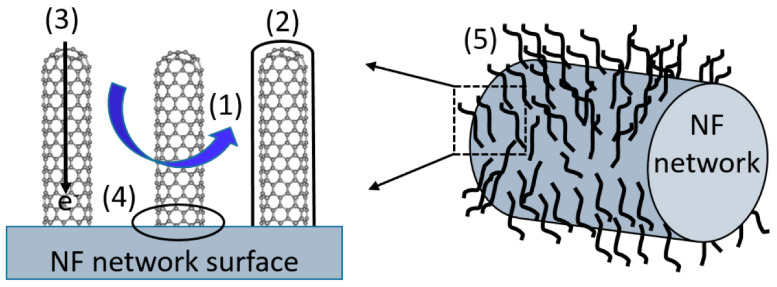
Illustration of advantages of the direct growing of CNTs on the NF collector.

The two-electrode test results are similar to those obtained from the three-electrode examination. Both the CV and GCD examinations of the two-electrode configuration showed high areal specific capacitance and good capacitance retention of the CNTs/NF electrode, indicating its high rate of performance due to its excellent electrochemical stability (The comparison of the areal specific capacitance of carbon-based nanomaterials/NF electrode is given in Table S2). The results can be contributed to directly growing the CNTs on the NF network surface, as shown in Figure 7, illustrating the advantages of the CNTs/NF electrode. The advantages of directly growing CNTs on NF network surface are summarized as: (1) The direct growing of CNTs avoided agglomeration, providing open microstructures for promoting the diffusion kinetics of the electrolyte. (2) Avoiding agglomeration issues allowed for the maximum utilization of the CNT surface area, maximizing the active electrochemical area. (3) The good conductivity and ballistic behavior of CNTs promoted CNTs as the highway for charge transportation, minimizing energy loss. (4) The binder-free interface provided the lowest interfacial resistance between the CNTs and the NF collector. (5) The binder-free CNTs/NF electrode led to the lightest weight of the CNTs. Additionally, compared with the traditional use of binders, the dispersing and mixing process for carbon-based materials and binders usually requires a long synthesis time at a high temperature, leading not only to the increasing weight of the electrode, but also resulting in the addition of carbon-based materials and a current collector at the coating interfaces, reducing the active area and charge transport paths, therefore degrading the electrochemical performance.

**Figure 8:**
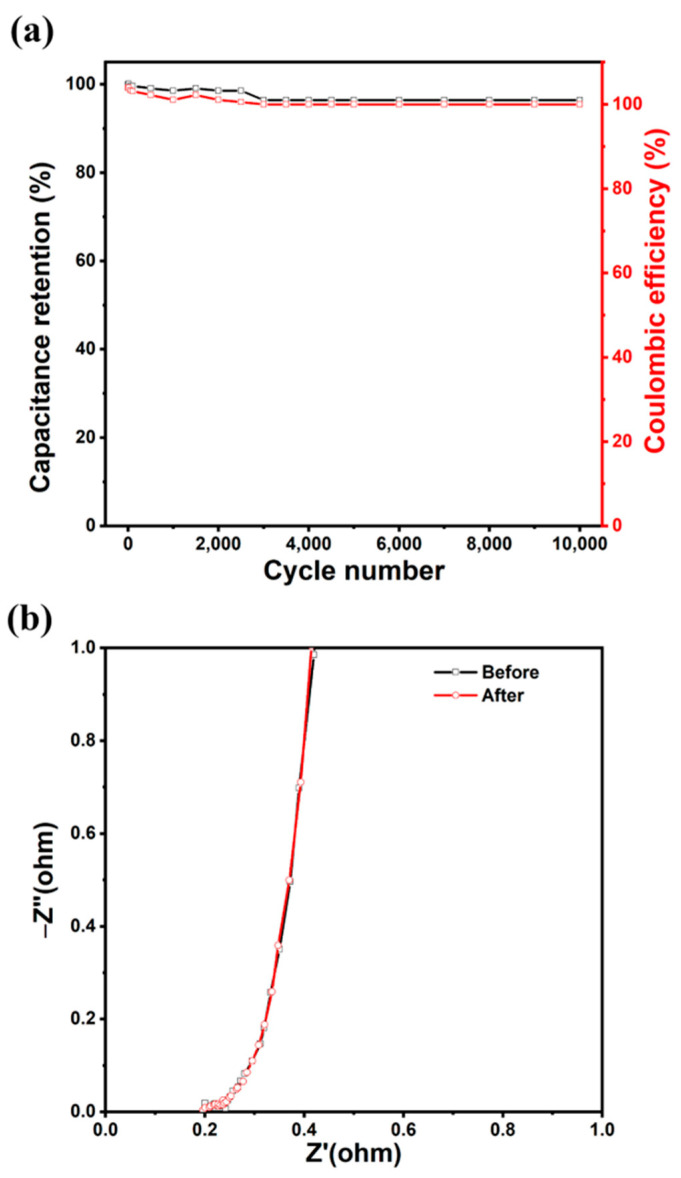
(**a**) Cycling performance, and (**b**) Nyquist plots before/after the cycling test of the CNTs/NF electrode.

The cycling stability of the CNTs/NF electrode was also measured using the GCD methods in the range of −1.0 to 0 V_SCE_ at a current density of 30 mA cm^−2^ for 10,000 cycles. Figure 8a shows the capacitance retention and Coulombic efficiency as a function of the cycle number. After 10,000 cycles, the CNTs/NF electrode displays a significantly long cycle life, with a capacitance retention of 96.4% of its initial specific capacitance. Additionally, the Coulombic efficiency is used to evaluate the efficiency of electron transfer within an electrochemical system, according to the output/input charge ratio of an SC. The obtained Coulombic efficiency shown in Figure 8a is well maintained at ~100%, with a slight change in capacitance, indicating that the CNTs/NF electrode exhibited not only good cycling stability, but also low internal resistance. To further characterize the cycling performance, EIS was also carried out to record the Nyquist plots before and after the total 10,000 cycles of the test, as shown in Figure 8b. It was observed that the impedance results, before and after the total 10,000 cycling test, were almost the same, and the EIS results corresponded with the Coulombic efficiency from the cycling stability test. The excellent cycle performance is attributed to the stable structure of the direct growing CNTs, with a strong attachment to the NF current collector, ensuring the good mechanical and electrical connection between the NF collector and the CNTs.

## 4. Conclusions

In summary, we have developed a facile and rapid CV strategy to obtain a self-converted Ni(OH)_2_ thin film coating on the NF surface. The Ni(OH)_2_ thin film could act as a suitable catalyst layer for directly growing CNTs on NF via a CVD process. It was found that the fabricated binder-free CNTs/NF electrode exhibited excellent performance for EDLC due to the open microstructure between CNTs, the small interfacial resistance between the CNTs and the NF collector, and the intrinsic properties of CNTs, including the high specific surface area and good conductivity. The direct growth of CNTs on NF also demonstrated the good mechanical and electrical connection during the cycling test. The CNTs/NF showed good specific capacitance values of 737.4 mF cm^−2^ in the three-electrode configuration and 319.1 mF cm^−2^ in the two-electrode configuration, at the current density of 1 mA cm^−2^. Moreover, the CNTs/NF displayed a good cycling stability of 96.4% capacitance retention after 10,000 cycles. This work provides a facile, rapid, and economical technique for fabricating a binder-free CNTs-based electrode for high performance SCs.

## Data Availability

Not applicable.

## Supplementary Materials

The following supporting information can be downloaded at: https://www.mdpi.com/article/10.3390/nano12213867/s1, Table S1: The C_S_ values and C_S_ retentions in *three-electrode* configuration, with increasing 100 times of CV scan rates and GCD current densities; Table S2: The C_S_ values and C_S_ retentions in *two-electrode* configuration, with increasing 100 times of CV scan rates and GCD current densities; Table S3: Comparison of the areal specific capacitance of carbon-based nanomaterials/NF electrode. References [28,29,30,31] were cited in the Supplementary Materials.

## References

[1] Service R.F. (2006). New Supercapacitor Promises to Pack more Electrical Punch. Science.

[2] Frackowiak E., Beguin F. (2001). Carbon materials for the electrochemical storage of energy in capacitors. Carbon.

[3] Laird E.A., Kuemmeth F., Steele G.A., Grove-Rasmussen K., Nygård J., Flensberg K., Kouwenhoven L.P. (2015). Quantum transport in carbon nanotubes. Rev. Mod. Phys..

[4] Anuratha K.S., Tsai Y.-H., Lin S.-Y., Chen I.-C., Sofer Z., Hsieh C.-K., Lin J.-Y. (2020). Graphitic nanofibers decorated with Ni_3_S_2_ interlaced nanosheets as efficient binder-free cathodes for hybrid supercapacitors. Appl. Surf. Sci..

[5] Chena T.Y., Vedhanarayanan B., Linb S.Y., Shaoc L.D., Soferd Z., Linb J.Y., Lina T.W. (2020). Electrodeposited NiSe on a forest of carbon nanotubes as a free-standing electrode for hybrid supercapacitors and overall water splitting. J. Colloid Interface Sci..

[6] Song H., Jeon H., Im D., Çakmakçı N., Shin K.-Y., Jeong Y. (2022). Free-standing carbon nanotube film for high efficiency monopole antenna. Carbon.

[7] Magrez A., Seo J.W., Smajda R., Mionić M., Forró L. (2010). Catalytic CVD Synthesis of Carbon Nanotubes: Towards High Yield and Low Temperature Growth. Materials.

[8] Manawi Y.M., Ihsanullah, Samara A., Al-Ansari T., Atieh M.A. (2018). A Review of Carbon Nanomaterials’ Synthesis via the Chemical Vapor Deposition (CVD) Method. Materials.

[9] Lee C.H., Johnson N., Drelich J., Yap Y.K. (2011). The performance of superhydrophobic and superoleophilic carbon nanotube meshes in water–oil filtration. Carbon.

[10] Zhang S., Zeng H.C. (2009). Self-Assembled Hollow Spheres of β-Ni(OH)_2_ and Their Derived Nanomaterials. Chem. Mater..

[11] Ash B., Nalajala V.S., Popuri A.K., Subbaiah T., Minakshi M. (2020). Perspectives on Nickel Hydroxide Electrodes Suitable for Rechargeable Batteries: Electrolytic vs. Chemical Synthesis Routes. Nanomaterials.

[12] Hall D.S., Lockwood D.J., Bock C., MacDougall B.R. (2015). Nickel hydroxides and related materials: A review of their structures, synthesis and properties. Proc. R. Soc. A Math. Phys. Eng. Sci..

[13] Li J., Liu Y., Cao W., Chen N. (2020). Rapid in situ growth of β-Ni(OH)_2_ nanosheet arrays on nickel foam as an integrated electrode for supercapacitors exhibiting high energy density. Dalton Trans..

[14] Ede S.R., Anantharaj S., Kumaran K.T., Mishra S., Kundu S. (2017). One step synthesis of Ni/Ni(OH)_2_ nano sheets (NSs) and their application in asymmetric supercapacitors. RSC Adv..

[15] Jang I.Y., Ogata H., Park K.C., Lee S.H., Park J.S., Jung Y.C., Kim Y.J., Kim Y.A., Endo M. (2010). Exposed Edge Planes of Cup-Stacked Carbon Nanotubes for an Electrochemical Capacitor. J. Phys. Chem. Lett..

[16] Randin J.-P., Yeager E. (1971). Differential Capacitance Study of Stress-Annealed Pyrolytic Graphite Electrodes. J. Electrochem. Soc..

[17] Iamprasertkun P., Hirunpinyopas W., Keerthi A., Wang B., Radha B., Bissett M.A., Dryfe R.A.W. (2019). Capacitance of Basal Plane and Edge-Oriented Highly Ordered Pyrolytic Graphite: Specific Ion Effects. J. Phys. Chem. Lett..

[18] Hata K., Futaba D.N., Mizuno K., Namai T., Yumura M., Iijima S. (2004). Water-Assisted Highly Efficient Synthesis of Impurity-Free Single-Walled Carbon Nanotubes. Science.

[19] Patole S., Alegaonkar P., Lee H.-C., Yoo J.-B. (2008). Optimization of water assisted chemical vapor deposition parameters for super growth of carbon nanotubes. Carbon.

[20] Smajda R., Andresen J.C., Duchamp M., Meunier R., Casimirius S., Hernádi K., Forró L., Magrez A. (2009). Synthesis and mechanical properties of carbon nanotubes produced by the water assisted CVD process. Phys. Status Solidi B.

[21] Shih Y.-J., Huang Y.-H., Huang C. (2018). Electrocatalytic ammonia oxidation over a nickel foam electrode: Role of Ni(OH)_2_(s)-NiOOH(s) nanocatalysts. Electrochim. Acta.

[22] Mansour A.N. (1994). Characterization of β-Ni(OH)_2_ by XPS. Surf. Sci. Spectra.

[23] Li H.B., Yu M.H., Wang F.X., Liu P., Liang Y., Xiao J., Tong Y.X., Yang G.W. (2013). Amorphous nickel hydroxide nanospheres with ultrahigh capacitance and energy density as electrochemical pseudocapacitor materials. Nat. Commun..

[24] Kostecki R., McLarnon F. (1997). Electrochemical and In Situ Raman Spectroscopic Characterization of Nickel Hydroxide Electrodes: I. Pure Nickel Hydroxide. J. Electrochem. Soc..

[25] Lin C.-H., Tsai C.-H., Tseng F.-G., Ma C.-C.M., Wu H.-C., Hsieh C.-K. (2017). Three-dimensional vertically aligned hybrid nanoarchitecture of two-dimensional molybdenum disulfide nanosheets anchored on directly grown one-dimensional carbon nanotubes for use as a counter electrode in dye-sensitized solar cells. J. Alloys Compd..

[26] Stoller M.D., Ruoff R.S. (2010). Best practice methods for determining an electrode material’s performance for ultracapacitors. Energy Environ. Sci..

[27] Peng C., Zhang S., Jewell D., Chen G.Z. (2008). Carbon nanotube and conducting polymer composites for supercapacitors. Prog. Nat. Sci..

[28] Ren G.F., Pan X., Bayne S., Fan Z.Y. (2014). Kilohertz ultrafast electrochemical supercapacitors based on perpendicularly-oriented graphene grown inside of nickel foam. Carbon.

[29] Chen J., Sheng K.X., Luo P.H., Li C., Shi G.Q. (2012). Graphene Hydrogels Deposited in Nickel Foams for High-Rate Electrochemical Capacitors. Adv. Mater..

[30] Sridhar D., Meunier J.-L., Omanovic S. (2019). Directly grown carbon nano-fibers on nickel foam as binder-free long-lasting supercapacitor electrodes. Mater. Chem. Phys..

[31] Yang J., Zhang E., Li X., Yu Y., Qu J., Yu Z.-Z. (2016). Direct Reduction of Graphene Oxide by Ni Foam as a High-Capacitance Supercapacitor Electrode. ACS Appl. Mater. Interfaces.

