# Supplementary material for: Facile and Rapid Electrochemical Conversion of Ni into Ni(OH)2 Thin Film as the Catalyst for Direct Growth of Carbon Nanotubes on Ni Foam for Supercapacitors
Source: Nanomaterials (Basel). 2022 Nov 2;12(21):3867. doi: 10.3390/nano12213867 (PMC9653567; doi:10.3390/nano12213867)
Supplement: Supplementary file 1 [file nanomaterials-12-03867-s001.zip › nanomaterials-1971839-supplementary.pdf]

nanomaterials

MDPI

Supplementary Information

Facile and Rapid Electrochemical Conversion of Ni into $Ni(OH)_2$ Thin Film as the Catalyst for Direct Growth of Carbon Nanotubes on Ni Foam for Supercapacitors

Sheng-Hung Kao [1], Krishnan Shanmugam Anuratha [2], Sung-Yen Wei [3], Jeng-Yu Lin [2,*] and Chien-Kuo Hsieh [1,*]

1 Department of Materials Engineering, Center for Plasma and Thin Film Technologies, Ming Chi University of Technology, New Taipei City 24301, Taiwan
2 Department of Chemical and Materials Engineering, Tunghai University, Taichung City 407224, Taiwan
3 R&D Lab, SulfurScience Technology Co. Ltd., New Taipei City 24301, Taiwan
* Correspondence: jylin@thu.edu.tw (J.-Y.L.); jack_hsieh@mail.mcut.edu.tw (C.-K.H.)

1. Raw data calculated from CV and GCD results using *two*- and *three*-electrode configurations

Table S1 shows the raw data of C_S values and C_S retentions calculated from CV and GCD examinations using *three-electrode* configuration. Both CV and GCD results maintained good capacitance retentions of 42.8 % and 60.4 %, respectively, with increasing 100 times of CV scan rates and GCD current densities. (852.5 mF cm^{-2} to 364.9 mF cm^{-2} at CV scan rates from 10 mA s^{-1} to 1000 mA s^{-1}, 737.4 mF cm^{-2} to 445.1 mF cm^{-2} at GCD current densities from 1 mA cm^{-2} to 100 mA cm^{-2}.)

Table S1. The C_S values and C_S retentions in *three-electrode* configuration, with increasing 100 times of CV scan rates and GCD current densities.

***Three-electrode* configuration**					
CV			GCD		
Scan rate (mV/s)	Cs (mF cm^{-2})	Retention (%)	Current density (mA cm^{-2})	Cs (mF cm^{-2})	Retention (%)
10	852.5	100.0	1	737.4	100.0
20	816.5	95.8	2	660.2	89.5
50	752.2	88.2	3	642.9	87.2
80	715.2	83.9	5	614.2	83.3
100	694.1	81.4	10	589.7	80.0
200	621.5	72.9	20	561.1	76.1
300	568.9	66.7	30	540.3	73.3
500	491.2	57.6	50	513.5	69.6
800	405.6	47.6	100	445.1	60.4
1000	364.9	42.8			

Table S2 shows the raw data of C_S values and C_S retentions calculated from CV and GCD examinations using *two-electrode* configuration. Both CV and GCD results maintained good capacitance retentions of 58.7 % and 49.0 %, respectively, with increasing 100 times of CV scan rates and GCD current densities. (265.1 mF cm^{-2} to 155.6 mF cm^{-2} at CV scan rates from 10 mA s^{-1} to 1000 mA s^{-1}, 319.1 mF cm^{-2} to 156.5 mF cm^{-2} at GCD current densities from 1 mA cm^{-2} to 100 mA cm^{-2}.)

Table S2. The Cs values and Cs retentions in *two-electrode* configuration, with increasing 100 times of CV scan rates and GCD current densities.

**Electrode**	**Synthesized method**	**Electro-lyte**	Specific aerial capacitance derived by GCD				Ref.
			Two-electrode		Three-electrode		
			Current density (mA cm^{-2})	Cs (mF cm^{-2})	Current density (mA cm^{-2})	Cs (mF cm^{-2})	
**Graphene/NF**	**Plasma en-hanced CVD**	**KOH**	1	~ 1.25			(1)
Graphene Hydro-gels/NF	Acid reduction	KOH	0.67	45.6			(2)
CNFs/NF	CVD	KOH	3	140.8			(3)
RGO/NF	Ni reduction controlled by pH	Na_2SO_4			0.5	323	(4)
CNTs/NF	CVD	KOH	1	319.1	1	737.4	This work

2. Comparison of the areal specific capacitance of carbon-based nanomaterials/NF electrode.

Due to the fact that the weight of direct growth of CNTs is an approximation of negligence. For this reason, the aerial specific capacitance is a more suitable quantity for relative comparison. Table S3 shows the comparison of the areal specific capacitance of carbon-based nanomaterials/NF electrode.

Table S3. Comparison of the areal specific capacitance of carbon-based nanomaterials/NF electrode.

***Two-electrode* configuration**					
**CV**			GCD		
**Scan rate (mV/s)**	**Cs (mF cm^{-2})**	**Retention (%)**	Current density (mA cm^{-2})	Cs (mF cm^{-2})	Retention (%)
10	265.1	100.0	1	319.1	100.0
20	262.7	99.1	2	290.6	91.1
50	251.7	94.9	3	285.2	89.4
80	245.1	92.5	5	273.5	85.7
100	241.6	91.1	10	257.8	80.8
200	226.8	85.6	20	241.2	75.6
300	214.7	81.0	30	228.3	71.5
500	194.4	73.3	50	206.1	64.6
800	169.5	63.9	100	156.5	49.0
1000	155.6	58.7			

Reference

1. Ren, G.F.; Pan, X.; Bayne, S.; Fan, Z.Y. Kilohertz ultrafast electrochemical supercapacitors based on perpendicularly-oriented graphene grown inside of nickel foam. *Carbon* **2014**, *71*, 94–101.
2. Chen, J.; Sheng, K.X.; Luo, P.H.; Li, C.; Shi, G.Q. Graphene Hydrogels Deposited in Nickel Foams for High-Rate Electrochemical Capacitors. *Adv. Mater.* 2012, *24*, 4569–4573.
3. Sridhar, D.; Meunier, J.-L.; Omanovic, S. Directly grown carbon nano-fibers on nickel foam as binder-free long-lasting supercapacitor electrodes. *Mater. Chem. Phys.* 2019, *223*, 434–440.
4. Yang, J.; Zhang, E.; Li, X.; Yu, Y.; Qu, J.; Yu, Z.-Z. Direct Reduction of Graphene Oxide by Ni Foam as a High-Capacitance Supercapacitor Electrode. *ACS Appl. Mater. Interfaces* 2016, *8*, 2297–2305.
